# Associations of geographic-based socioeconomic factors and HPV vaccination among male and female children in five US states

**DOI:** 10.1186/s12889-024-18206-5

**Published:** 2024-03-05

**Authors:** Serena Xiong, Sarah Humble, Alan Barnette, Heather Brandt, Vetta Thompson, Lisa M. Klesges, Michelle I. Silver

**Affiliations:** 1 Department of Family Medicine and Community Health, University of Minnesota Medical School, 717 Delaware St SE, Suite 166, 55414 Minneapolis, MN USA; 2 Department of Surgery, Washington University School of Medicine, 600 S Taylor Avenue, 63110 St. Louis, MO USA; 3 Saint Francis Medical Center, 211 St. Francis Drive, 63703 Cape Girardeau, MO USA; 4 HPV Cancer Prevention Program, St. Jude Children’s Research Hospital, 262 Danny Thomas Place, 38105-3678 Memphis, TN USA; 5 Barnes-Jewish Hospital, Alvin J. Siteman Cancer Center, Washington University School of Medicine, 63110 St. Louis, MO USA; 6 Department of Medicine and Pediatrics, Washington University School of Medicine, Washington University in St. Louis, 63110 St. Louis, MO USA

**Keywords:** Human papillomavirus (HPV), HPV vaccination, Geographic-based factors, Generalized estimation equation models

## Abstract

**Background:**

We assessed whether five geographic-based socioeconomic factors (medically underserved area (MUA); healthcare provider shortage area (HPSA); persistent poverty; persistent child poverty; and social vulnerability index (SVI)) were associated with the odds of HPV vaccination initiation, series completion, and parental vaccine hesitancy, and whether the observed relationships varied by gender of the child.

**Methods:**

An online panel service, administered through Qualtrics®, was used to recruit parents of adolescents 9–17 years of age to complete a one-time survey in 2021. Coverage of the panel included five US states: Arkansas, Mississippi, Missouri, Tennessee, and Southern Illinois. Generalized estimating equation (GEE) models were used to assess population-level associations between five geographic-based socioeconomic factors (MUA; HPSA; persistent poverty; persistent child poverty; and SVI) and three HPV vaccination outcomes (initiation, series completion, and hesitancy). All GEE models were adjusted for age of child and clustering at the state level.

**Results:**

Analyses were conducted using responses from 926 parents about their oldest child in the target age range (9–17 years). The analytic sample consisted of 471 male children and 438 female children across the five states. In adjusted GEE models, persistent child poverty and HPSA were negatively associated with HPV vaccination initiation and series completion among female children, respectively. Among male children, high social vulnerability was negatively associated with HPV vaccine series completion. Additionally, persistent poverty and high social vulnerability were negatively associated with HPV vaccine hesitancy in male children.

**Conclusions:**

The results of this cross-sectional study suggest that geographic-based socioeconomic factors, particularly, HPSA, persistent poverty, and SVI, should be considered when implementing efforts to increase HPV vaccine coverage for adolescents. The approaches to targeting these geographic factors should also be evaluated in future studies to determine if they need to be tailored for male and female children.

## Background

Human papillomavirus (HPV) vaccination has significantly reduced HPV infections among US adolescents since its introduction in 2006 [1]. Ongoing post-market surveillance studies have also demonstrated its efficacy and effectiveness in reducing invasive cervical cancer cases [2]. For preventing HPV infections and HPV-associated cancers (e.g., cervical, nasopharyngeal, oral), vaccines remain an effective and economical strategy [3]. Currently, children in the US are recommended to receive HPV vaccines as soon as they are 9 years old, with routine vaccinations recommended for 11 or 12 year olds [4].

Before the coronavirus pandemic of 2019 (COVID-19), HPV vaccination coverage in US children was relatively high. Estimates from the National Immunization Survey conducted in 2019 found an HPV vaccination coverage (≥ 1 dose of HPV vaccine) as high as 71.5% for all adolescents [5]. A pre-pandemic study using nationally representative data from the 2017–2020 National Health and Nutrition Examination Survey also reported similar coverage rates [6]. Due to restricted and declining routine preventive care since the COVID-19 pandemic, HPV vaccination among children has likely also declined.

Studies conducted before the pandemic indicated disparities in adolescent HPV vaccination coverage based on race/ethnicity, gender, and geographic location [7–9]. In a 2019 systematic review and meta-analysis examining racial and ethnic disparities in HPV vaccination, it was found that there are no overall differences in HPV vaccination initiation rates. However, the study revealed that racial and ethnic minorities are less likely than their White counterparts to complete the full series of the vaccine [9]. Since the initial recommendations for the HPV vaccine were only for females, though were eventually expanded to include males in 2009, this could have potentially led to unequal vaccination rates among US male and female adolescents after the introduction of the vaccine [10]. As reported in data from 2018, it was observed that 70% of females and 66% of males between the ages of 13 and 17 had received at least one dose of the vaccine. Furthermore, 53% of females and 49% of males had completed the full series of the vaccine [11]. However, recent trend data from the 2015 to 2020 National Immunization Survey-Teen (NIS-Teen), indicates a narrowing of the differences by gender over time [12].

Disparities in HPV vaccination rates have also been observed based on geographical locations [8, 13–15]. Data from the 2016 NIS-Teen indicated that adolescents in the Western US states (e.g., California, Washington) were more likely to have received the vaccine in comparison to those in the South (e.g., Texas, Louisiana) [16]. Similarly, data from 2013 NIS-Teen revealed that several Southern and Midwestern states continue to have lower HPV vaccination rates for both adolescent females and males, lagging behind Western and Northeastern states [17]. The disparities in HPV vaccination based on geography underscore the importance of gathering additional data and maintaining continuous surveillance of adolescent vaccination rates within regions with lower coverage and initiation rates, such as Midwestern and Southern states (e.g., Missouri, Mississippi).

Further complicating and adding to the heterogeneity of HPV vaccination rates are area-level characteristics, such as provider shortage areas [18]. An illustration of the heterogeneity in HPV vaccination coverage can be seen in states like Illinois, where the overall coverage rate may be moderately high. However, disparities exist between urban and rural areas within the state, with rural areas more likely to face shortages of healthcare providers. As shown in a 2023 study by Boakye and colleagues, adolescents living in rural areas in Illinois were 38% and 24% less likely to initiate or complete the HPV vaccine, respectively, compared to their urban counterparts [19]. Other geographic-based socioeconomic factors such as area-level poverty (e.g., USDA-defined persistent poverty, social vulnerability index) and health service region characteristics (e.g., medically underserved areas, healthcare provider shortage areas) have also been shown to impact adolescent HPV vaccinations [5, 20, 21]. A recent systematic review found that higher poverty levels are generally associated with higher initiation rates; however, a county-level shortage of providers was also associated with a decrease in HPV vaccination initiation [18]. The potentially opposing effects of these associations warrant a more in-depth investigation into the varied types of geographic-based socioeconomic factors and their impact on HPV vaccination coverage. Further, such investigations will be useful for identifying areas for systems-level intervention efforts.

In light of the *Healthy People 2030* goals and initiatives (i.e., DOSE HPV: Development of Systems and Education for HPV Vaccination) to increase HPV vaccination rates via systematic approaches, research is needed to better understand geographic-based socioeconomic factors and their impact on HPV vaccination among US children [22]. As HPV vaccination coverage continues to vary across the US, more population health approaches (e.g., multilevel methods) are needed to better tailor prevention efforts to areas of high need. The current study uses generalized estimating equation (GEE) models, a multilevel modeling approach, to assess geographic-based factors and their associations with HPV vaccine uptake among male and female children across five US states.

## Methods

### Study sample, recruitment, and procedures

An online panel service, administered through Qualtrics®, was used to recruit parents of adolescents 9–17 years of age to complete a one-time survey in July 2021. Participants were eligible if they had at least one child within the target age range and lived in one of the following five states: Arkansas, Mississippi, Missouri, Tennessee, or select counties in Southern Illinois. The full study procedures and survey measures are documented in a prior publication [23]. A total of 926 parents and their oldest child in the target age range were recruited for the study. This study was approved by the Washington University in St. Louis Institutional Review Board (IRB) (Study#202106066).

### Geographic-based socioeconomic measures

Participants’ geographic location was determined using their self-reported home ZIP code. These ZIP codes were spatially joined to the corresponding county and shortage designation areas using ArcMap 10.6.1 (ESRI, Redlands, CA). Shortage designation areas were determined based on the geographic boundaries defined by the Health Resources and Service Administration (HRSA) [24]. Residing in a medically underserved area (MUA) was defined as living in a ZIP code within a geographic area that had a shortage of primary care health services. Residing in a healthcare provider shortage area (HPSA) was defined as living in a ZIP code within a geographic area that had a shortage of primary care providers. County-level socioeconomic measures included measures of persistent poverty for all residents and one for children under the age of 18 [25] the social vulnerability index (SVI; Centers for Disease Control and Prevention, CDC) [26]. Persistent poverty counties are counties where greater than 20% of the residents were poor in each of the censuses from 1980 to 2000 as well as the 2007–2011 American Community Survey five-year average. Persistent child poverty counties followed the same definition, but only among children under 18 years old. The SVI of each county indicates the relative vulnerability of a county after a disaster and includes measures such as socioeconomic status, household composition, disability, minority status, language, housing type, and transportation. For this analysis, we used the CDC’s SVI measure [26] and dichotomized participants living in counties with a SVI greater or equal to 0.7 to be living in high vulnerability counties, as consistent with a previous study [27].

### Outcome measures

The primary outcomes in this study were HPV vaccination initiation, HPV vaccine series completion, and HPV vaccine hesitancy. A child was considered to have initiated the HPV vaccine series if their parent answered “yes” to the question “Has [oldest child] been vaccinated against HPV?” HPV series completion was determined based on follow up questions about the age of the child at first vaccination and the number of shots in the series that they had completed. According to the CDC’s recommended schedule for HPV vaccination in adolescents, if a child receives their first dose before the age of 15, their series is considered complete after two doses. However, for children receiving their first dose at 15 or after, series completion occurs after three doses [28]. HPV vaccine hesitancy was assessed using an adapted version of the 5-item Vaccine Hesitancy Scale (Cronbach’s alpha = 0.81) [23, 29]. Parents answered their level of agreement to each of these five items: “The HPV vaccine is effective”, “The HPV vaccine is beneficial for my adolescent”, “I do/did what my adolescent’s healthcare provider recommends about HPV vaccination”, and “I am concerned about serious side effects of the HPV vaccine” on a 4-point Likert-type scale (strongly agree to strongly disagree). Items were coded so that higher scores indicated increasing hesitancy to HPV vaccination. In analysis, the composite score was dichotomized, as modeled in previous studies [23, 29], with a score greater than three indicating hesitancy.

### Data analysis

Descriptive frequencies were calculated for all geographic-based socioeconomic measures and reported by state. Given the nested structure of our data (e.g., individuals nested within states), we used GEE models to assess population-level associations between geographic-based socioeconomic factors and HPV vaccination outcomes (initiation, series completion, and hesitancy). Such a strategy allowed us to account for community-level and individual-level covariates within a single analysis. These population average models, then, produce odds ratios that are interpreted in a similar way to standard logistic regression models. The parameter estimates reflect the impact of each predictor, averaged across all states. An exchangeable covariance structure was used with robust estimates in both unadjusted and adjusted GEE models Separate equations were performed for each outcome (i.e., initiation, series completion, and hesitancy), with stratification based on gender for each geographic-based socioeconomic predictor. All GEE models, therefore, were not mutually adjusted for the five geographic-based socioeconomic predictors. SAS version 9.4 (SAS Institute, Cary, NC) was used to conduct all analyses.

## Results

### Descriptive statistics

A total of 471 male and 438 female children were included in the analysis (*n* = 909). Sociodemographic characteristics of both parents and children are shown in Table 1. The mean age of all children was 13.81 years old (SD = 2.49). Among parents, the largest percentage fell within the age range of 40 to 45 years (27.7%). A significant majority identified as female (78.2%), non-Hispanic white (76.0%), and 42.4% had either attended some college or completed an associate’s degree. The majority of parents were employed, constituting 64.2% of the total. On average, parent participants reported either living in urban (*n* = 340) or small rural geographic areas (*n* = 383). Across all outcomes, a greater proportion of female children reported HPV vaccination initiation, series completion, and their parents were less hesitant (data not shown).

**Table 1:** Sociodemographic characteristics of parent and children participants (*n* = 909)

	N	%
Age in years of parent	906	
25–29	37	4.08
30–34	172	18.98
35–39	228	25.17
40–45	251	27.70
45+	218	24.06
Age in years of child	909	
9–12	270	29.70
13–17	639	70.30
Gender of parent	906	
Male	195	21.52
Female	708	78.15
Genderqueer/non-binary	3	0.33
Gender of child	909	
Male	471	51.82
Female	438	48.18
Race of parent	900	
Non-Hispanic White	684	76.00
Non-Hispanic Black	125	13.89
Some other race, including multiple	91	10.11
Participants by state	909	
AR	173	19.03
IL	67	7.37
MO	287	31.57
MS	57	6.27
TN	325	35.75
Self-described geographic area	894	
Urban	340	38.03
Large rural	171	19.13
Small rural/Isolated	383	42.84
Parent level of educational attainment	904	
High school graduate or equivalency (GED) or less	247	27.32
Some college/Associate’s degree or technical school certification	383	42.37
College graduate, graduate degree or more	274	30.31
Parent employment status	902	
Employed	579	64.19
Stay-at-home/Homemaker	179	19.84
Other	144	15.96

Distributions of geographic-based socioeconomic variables across the five states are presented in Table 2. Mississippi had the greatest proportions of children living in MUA (84.21%), HPSA (35.09%), persistent poverty (33.33%), persistent child poverty (52.63%), and high SVI (54.39%) areas. Missouri had the lowest proportions of children living in MUA (27.55%) and HPSA (1.02%) areas. Illinois had the lowest proportions of children living in persistent child poverty (11.76%) and high SVI (10.29%) areas. Tennessee reported the lowest proportion of children living in persistent poverty (3.31%).

**Table 2:** Distributions of geographic-based socioeconomic variables among adolescents living across five states (*n* = 909)

Geographic-based socioeconomic factors	Arkansas (*n* = 173)		Illinois (*n* = 67)		Missouri (*n* = 287)		Mississippi (*n* = 57)		Tennessee (*n* = 325)	
	N	%	N	%	N	%	N	%	N	%
Medically Underserved Area (MUA)										
Non-MUA	78	45.09	43	64.18	208	72.47	9	15.79	124	38.15
MUA	95	54.91	24	35.82	79	27.53	48	84.21	201	61.85
Healthcare Provider Shortage Area (HPSA)										
Non-HPSA	163	94.22	49	73.13	285	99.30	37	64.91	299	92.00
HPSA	10	5.78	18	26.87	2	0.70	20	35.09	26	8.00
Persistent poverty										
Not persistent poverty	154	89.02	63	94.03	260	90.59	38	66.67	315	96.92
Persistent poverty	19	10.98	4	5.97	27	9.41	19	33.33	10	3.08
Persistent child poverty										
Not persistent child poverty	136	78.61	59	88.06	247	86.06	27	47.37	248	76.31
Persistent child poverty	37	21.39	8	11.94	40	13.94	30	52.63	77	23.69
Social Vulnerability Index										
Low vulnerability^1^	91	52.60	60	89.55	236	82.23	26	45.61	224	68.92
High vulnerability^2^	82	47.40	7	10.45	51	17.77	31	54.39	101	31.08

^1^ Low vulnerability measured as Social Vulnerability Index < 0.7

^2^ High vulnerability measured as Social Vulnerability Index ≥ 0.7

### Multi-level analyses

In unadjusted GEE analyses (Table 3), HPSA was associated with HPV vaccine series completion but only for females. Adolescent females living in HPSA areas were less likely to have completed the HPV vaccine series compared to their counterparts in non-HPSA areas (OR_unadj_. = 0.61, 95% CI = 0.52–0.71). For HPV vaccination initiation, persistent child poverty was a negative predictor but only for females (OR_unadj_.= 0.58, 95% CI = 0.35–0.95). Meanwhile, persistent poverty and high social vulnerability were negatively associated with HPV vaccine hesitancy in males (OR_unadj_. = 0.46, 95% CI = 0.26–0.84; OR_unadj_. = 0.45, 95% CI = 0.43–0.47, respectively). After adjusting for the age of the child, all associations remained statistically significant (Table 4).

**Table 3:** Unadjusted population average (GEE) models^1^ of HPV Vaccination Outcomes (**Initiation, Series Completion, Hesitancy**) and geographic-based socioeconomic factors by male and female child (*n* = 909)

Geographic-based socioeconomic factors	Initiation		Series Completion		Hesitancy	
	Males (*n* = 471)	Females (*n* = 438)	Males (*n* = 471)	Females (*n* = 438)	Males (*n* = 471)	Females (*n* = 438)
	OR (95% CI)	OR (95% CI)	OR (95% CI)	OR (95% CI)	OR (95% CI)	OR (95% CI)
Medically Underserved Area (MUA) vs. Non-MUA	0.98 (0.78–1.24)	0.99 (0.67–1.47)	1.02 (0.75–1.39)	1.05 (0.76–1.45)	1.19 (0.78–1.80)	1.20 (0.68–2.10)
Healthcare Provider Shortage Area (HPSA) vs. non-HPSA	1.14 (0.67–1.94)	0.82 (0.47–1.42)	1.48 (0.89–2.46)	0.61 (0.52–0.71)*	0.80 (0.45–1.41)	1.13 (0.63–2.04)
Persistent poverty vs. none	2.00 (0.73–5.48)	1.31 (0.52–3.26)	1.92 (0.59–6.19)	1.04 (0.35–3.10)	0.46 (0.26–0.84)*	1.07 (0.54–2.11)
Persistent child poverty vs. none	0.95 (0.42–2.13)	0.58 (0.35–0.95)*	1.42 (0.60–3.34)	0.63 (0.34–1.17)	0.83 (0.62–1.11)	1.10 (0.67–1.79)
High social^2^ vulnerability vs. low^3^	0.89 (0.55–1.45)	0.95 (0.62–1.48)	1.32 (0.64–2.71)	1.01 (0.58–1.75)	0.45 (0.43–0.47)*	1.23 (0.97–1.56)

OR = odds ratio; 95% CI = 95% confidence interval

* statistically significant at alpha level of < 0.05

^1^ Population average model/GEE– clustered on state

^2^ High vulnerability measured as Social Vulnerability Index ≥ 0.7

^3^ Low vulnerability measured as Social Vulnerability Index < 0.7

**Table 4:** Adjusted^#^ population average (GEE) models^1^ of HPV Vaccination Outcomes (**Initiation, Series Completion, Hesitancy**) and geographic-based socioeconomic factors by male and female child (*n* = 909)

Geographic-based socioeconomic factors	Initiation		Series Completion		Hesitancy	
	Males (*n* = 471)	Females (*n* = 438)	Males (*n* = 471)	Females (*n* = 438)	Males (*n* = 471)	Females (*n* = 438)
	aOR (95% CI)	aOR (95% CI)	aOR (95% CI)	aOR (95% CI)	aOR (95% CI)	aOR (95% CI)
Medically Underserved Area (MUA) vs. Non-MUA	0.98 (0.81–1.18)	1.17 (0.89–1.55)	1.14 (0.79–1.64)	1.10 (0.79–1.53)	1.19 (0.78–1.82)	1.22 (0.70–2.13)
Healthcare Provider Shortage Area (HPSA) vs. non-HPSA	1.06 (0.58–1.94)	1.02 (0.65–1.59)	1.64 (0.94–2.88)	0.65 (0.47–0.91)*	0.80 (0.45–1.41)	1.17 (0.67–2.05)
Persistent poverty vs. none	1.80 (0.71–4.56)	1.35 (0.56–3.26)	1.71 (0.52–5.65)	1.32 (0.53–3.33)	0.46 (0.25–0.87)*	1.10 (0.52–2.33)
Persistent child poverty vs. none	0.90 (0.43–1.85)	0.58 (0.42–0.80)*	1.43 (0.59–3.47)	0.63 (0.39–1.01)	0.83 (0.61–1.13)	1.11 (0.64–1.92)
High social^3^ vulnerability vs. low^4^	0.88 (0.59–1.30)	0.99 (0.72–1.37)	0.55 (0.42–0.50)*	1.23 (0.93–1.63)	0.46 (0.42–0.50)*	1.23 (0.93–1.63)

aOR = adjusted odds ratio; 95% CI = 95% confidence interval

^#^ adjusted for age of child

* statistically significant at alpha level of < 0.05

^1^ Population average model/GEE– clustered on state

^2^ High vulnerability measured as Social Vulnerability Index ≥ 0.7

^3^ Low vulnerability measured as Social Vulnerability Index < 0.7

## Discussion

Identifying the types of geographic-based socioeconomic factors that affect HPV vaccination in US children can assist in more tailored prevention and control measures, especially after a decline in preventive care services such as that resulting from the COVID-19 pandemic.

In our study, we found that persistent child poverty is a negative predictor of HPV vaccination initiation among female children. This finding is corroborated in several studies, where area-level poverty was associated with lower odds of HPV vaccination initiation among females [30, 31]. We also found that female children living in HPSA areas were less likely to complete their HPV vaccination series when compared to their non-HPSA counterparts. This finding is also consistent with other studies that have observed decreased HPV vaccination coverage in areas with limited access to healthcare providers [31]. We found no significant associations between geographic-based socioeconomic factors and HPV vaccine hesitancy among females within our study. This discovery aligns with a recently published cross-sectional study examining the prevalence of HPV hesitancy in US adolescents, which found that geographic-based socioeconomic factors (e.g., country region and metropolitan statistical areas) were not associated with vaccine hesitancy [32]. Furthermore, previous qualitative and quantitative research has demonstrated the importance of a provider’s role in addressing parental hesitancy of HPV vaccines. It may be that the frequency and quality of provider recommendations, rather than the availability of providers (e.g., healthcare provider shortages), have a stronger or more direct influence on addressing vaccine hesitancy among parents [33, 34]. These results emphasize the urgent need to address both area-level factors, such as persistent child poverty and healthcare provider shortages, and individual-level factors (e.g., provider recommendation) in order to increase HPV vaccination rates and coverage among adolescent females.

Similarly, male children living in high SVI areas were less likely to complete their HPV vaccines series. This finding is in contrast to other studies, where area-based poverty was associated with higher odds of vaccine series completion [18, 20]. We also found in our study that parents living in more highly socially vulnerable (e.g., high SVI areas) and persistent poverty spots are less likely to be vaccine hesitant with their boys; however, these associations were not observed in female children. One explanation for this inverse relationship could be a lack of HPV vaccine awareness on the parents’ part. It is possible that because HPV vaccinations are less heavily promoted for adolescent boys, parents may be unaware that they are available to them, and therefore are less hesitant to administer it [35, 36]. In order to gain a deeper understanding of the factors contributing to HPV vaccination hesitancy among adolescent males and to identify potential intervention strategies, further research utilizing mixed methods approaches, including qualitative methods, is necessary. This research should involve a diverse group of key stakeholders, including healthcare providers, parents and caregivers, and adolescent males themselves. By engaging these various perspectives, more nuanced insights can be gleaned as to the underlying causes of vaccine hesitancy, or the lack thereof, among parents of adolescent boys. Such information can lead to the development and implementation of more targeted interventions tailored to address the unique concerns and motivations of adolescent boys, thereby improving HPV vaccination uptake among this population.

Overall, these research results reveal differential effects of geographic-based socioeconomic factors on HPV vaccination among US female and male children. This study shows that health service region characteristics (e.g., HPSA) and area-level poverty (e.g., persistent child poverty) are associated with lower vaccination initiation and series completion among girls, respectively. Whereas area-level poverty (e.g., persistent poverty, high social vulnerability) are linked to lower vaccination series completion and vaccine hesitancy among boys. The distinct patterns of geographic-based socioeconomic factors across different outcomes of HPV vaccination (such as initiation, series completion, and hesitancy) among adolescent females and males highlight the necessity for targeted and multi-level strategies in promoting and implementing HPV vaccination. Implementing strategies that address multiple levels of influence, including clinics/practices, healthcare providers, and policies, can be instrumental in mitigating structural barriers, particularly in areas characterized by high poverty levels such as rural communities. Such comprehensive efforts have been shown to effectively increase uptake of HPV vaccination among both genders [37]. Further investigation is also needed to better understand the role of geographic-based socioeconomic factors and their potential interactions with individual factors (e.g., providers) on vaccine hesitancy among all eligible children. Given the current COVID-19 pandemic, it is crucial to prioritize examining uptake and addressing hesitancy to mitigate the pandemic’s impact on already sub-optimal HPV vaccine rates.

### Limitations

Although the current study adds to our understanding of how geographic-based socioeconomic factors impact HPV vaccination in male and female children, several limitations should be noted. The most notable limitation is the small sample size within certain states (Illinois, Mississippi) across geographic-based socioeconomic factors. Small area estimation techniques can be employed in future studies to create more granular and reliable estimates [38]. Similar to previous cross-sectional studies conducted during the ongoing COVID-19 pandemic our study examined vaccine uptake and hesitancy within this unique context, recognizing that parental responses may be influenced differently compared to pre-pandemic circumstances [39, 40]. Further, this study relied on parental self-reporting for their children’s vaccination status, introducing a potential limitation in accurately assessing the true extent of vaccination uptake. Moreover, the current analysis did not assess for interactions between area-level and individual-level characteristics (e.g., race/ethnicity, educational level, insurance status, provider recommendation). Such cross-level interactions may help to elucidate potential ecological fallacies and allow for opportunity to explore how area-level effects differ across various individual-level variables. The gathered information can be utilized to guide programmatic efforts to focus targeted messaging and campaigns that aim to increase HPV vaccination rates among specific populations and socioeconomic backgrounds in various geographic regions.

## Conclusions

The results of this study suggest that geographic-based socioeconomic factors, particularly, HPSA, persistent poverty, and SVI, should be considered when implementing efforts to increase HPV vaccine coverage for adolescents. The approaches to targeting these geographic factors should also be evaluated in future studies to determine if they need to be tailored for male and female children. It is crucial to further investigate these discrepancies and develop interventions that enhance HPV vaccination awareness, address hesitancy, and improve uptake among all eligible children. Future research should involve larger and more representative sample sizes and explore cross-level interactions to better understand the complexities of the relationship between geographic-based socioeconomic factors and adolescent HPV vaccination.

## Data Availability

The datasets used and/or analyzed during the current study are available from the corresponding author on reasonable request.

## Abbreviations

Adj.: Adjusted
CDC: Centers for Disease Control and Prevention
CI: Confidence interval
COVID-19: Coronavirus disease of 2019
GEE: Generalized estimating equation
HPSA: Healthcare provider shortage area
HPV: Human Papillomavirus
HRSA: Health Resources and Service Administration
IRB: Institutional Review Board
MUA: Medically underserved area
NIS-Teen: National Immunization Survey-Teen
OR: Odds ratio
SD: Standard deviation
SVI: Social vulnerability index
Unadj.: Unadjusted
USDA: US Department of Agriculture
